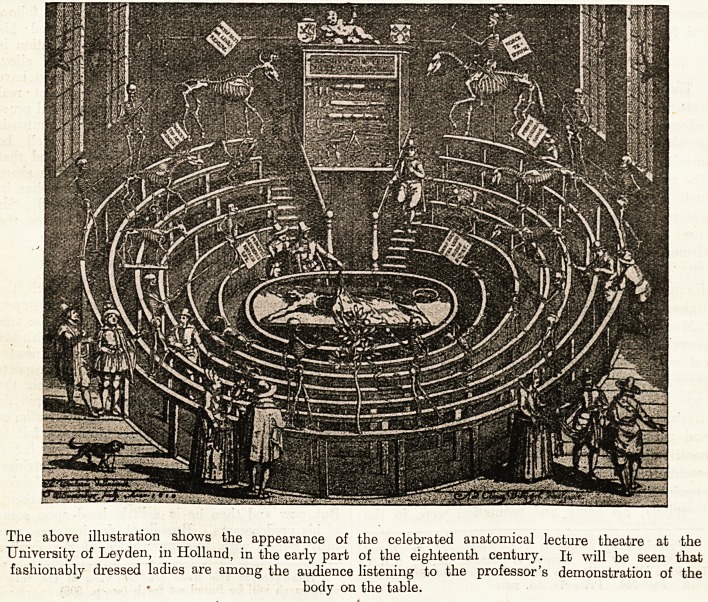# An Early Eighteenth-Century Anatomy Lecture Room

**Published:** 1916-09-02

**Authors:** 


### AN EARLY EIGHTEENTH-CENTURY ANATOMY LECTURE ROOM.

The above illustration shows the appearance of the celebrated anatomical lecture theatre at the University of Leyden, in Holland, in the early part of the eighteenth century. It will be seen that fashionably dressed ladies are among the audience listening to the professor's demonstration of the body on the table.

## Figures and Tables

**Figure f1:**